# Challenges and opportunities for designing clinical trials for antibody mediated rejection

**DOI:** 10.3389/frtra.2024.1389005

**Published:** 2024-06-12

**Authors:** Suryanarayanan Balakrishnan, Mariam P. Alexander, Carrie Schinstock

**Affiliations:** ^1^ Division of Hypertension and Nephrology, Department of Internal Medicine, Mayo Clinic, Rochester, MN, United States; ^2^ Department of Laboratory Medicine and Pathology, Mayo Clinic, Rochester, MN, United States

**Keywords:** antibody mediated rejection (ABMR), clinical trials, donor specific antibody (DSA), kidney transplant, allograft survival, surrogate endpoint

## Abstract

Significant progress has been made in kidney transplantation, with 1-year graft survival nearing 95%. However, long-term allograft survival remains suboptimal, with a 10-year overall graft survival rate of only 53.6% for deceased donor transplant recipients. Chronic active antibody-mediated rejection (ABMR) is a leading cause of death-censored graft loss, yet no therapy has demonstrated efficacy in large, randomized trials, despite substantial investment from pharmaceutical companies. Several clinical trials aimed to treat chronic ABMR in the past decade have yielded disappointing results or were prematurely terminated, attributed to factors including incomplete understanding of disease mechanisms, heterogeneous patient populations with comorbidities, slow disease progression, and limited patient numbers. This review aims to discuss opportunities for improving retrospective and prospective studies of ABMR, focusing on addressing heterogeneity, outcome measurement, and strategies to enhance patient enrollment to inform study design, data collection, and reporting.

## Introduction

Major advances have been made in kidney transplantation. One year graft survival now approaches 95% (1), but long term allograft survival remains suboptimal despite gradual improvements prospectively. The 10 year overall graft survival rate is only 53.6% for deceased donor transplant recipients transplanted from 2008 to 2011 (2). A leading contributor to death censored graft loss is chronic active antibody mediated rejection (ABMR) (3, 4), and no therapy has shown effectiveness in large, randomized trials. This is especially frustrating given the increasing investment that large pharmaceutical companies have dedicated to this important condition.

In the last decade, several important clinical trials in this field have shown disappointing results or prematurely terminated (5–7). The reasons include our incomplete understanding of the mechanisms driving progressive disease, a complicated heterogenous patient population, and slowly progressive disease prospective and retrospective study design must take these issues into consideration. To complicate this field, ABMR and microvascular inflammation in the absence of HLA DSA has been idenfied and acknowledge in the most recent iteration of the Banff classification (8). The aim of this review is to discuss opportunities to improve our retrospective and prospective studies of ABMR with the goal of finding an effective therapy to improve long-term outcomes. Our focus will be on approaches to reduce heterogeniety, considerations for outcome measurement, and strategies to increase patient enrollment to inform study design, data collection, and reporting.

## Minimizing patient heterogeneity in clinical trials

### Consider the varied clinical phenotypes of ABMR

Antibody-mediated rejection (ABMR) is diagnosed histologically by microvascular inflammation (glomerulitis and peritubular capillaritis) with or without C4d positivity (8). Evidence of HLA DSA is required, but C4d positivity or validated ABMR-associated molecular transcripts can fulfill these criteria if DSA is absent. Chronic ABMR is indicated by histologic features of chronic glomerulopathy (Banff cg score >0). Notably, the latest Banff classification recognizes a subgroup with DSA-negative, C4d-negative microvascular inflammation (8). Relying solely on histologic criteria for ABMR diagnosis has limitations due to potential variations in mechanisms and prognosis despite similar histologic features. Antibodies may be preexisting (i.e., pretransplant DSA) or develop post-transplant due to HLA mismatch (i.e., *de novo* DSA). ABMR can also be subclinical, detected via surveillance biopsy, or identified during allograft dysfunction on indication biopsy.

Early post-transplant increases in DSA can be driven by memory B cell responses, while persistently elevated DSA may be the result of constant secretion of alloantibody by the plasma cells. Another critical point to understand is that ABMR is a chronic progressive disease process, and the allograft survival depends on the time of detection. All these factors must be considered when defining patient inclusion criteria, determining the sample size, and developing a management strategy because outcomes differ among these groups. For example, graft survival 8 years after ABMR diagnosis was 63% among patients with preexisting DSA compared to only 34% among patients with *de novo* DSA (9). Furthermore, the median time to graft loss among patients with ABMR and *de novo* DSA who present with graft dysfunction is 3.3 years compared to 8.3 years if the ABMR is subclinical (10). Thus, there is a risk for underpowering a study or not finding therapeutic efficacy if original graft loss projections are based on patients with ABMR from *de novo* DSA and allograft dysfunction, but patients with preexisting DSA and ABMR detected on surveillance biopsy are eventually studied.

A consensus conference sponsored by the Transplantation Society considered the limitations of using histology alone for the diagnosis of ABMR and a framework of the clinical phenotypes of ABMR was constructed (11). The intent of this framework was to influence future ABMR study design and patient management by addressing the heterogeneity in clinical trials Table 1.

**Table 1:** Framework of ABMR clinical phenotypes.

Timing	Donor specific antibody		Histology	Clinical presentation
Hyperactive rejection (hours post-transplant)	Preexisting		Diffuse inflammation, necrosis, and thrombotic microangiopathy	Abrupt graft loss
Early active (<30 days post-transplant)	Preexisting (or patient is Non immunologically naïve with history of sensitizing events including pregnancy, transplant, or blood transfusion)	Can have similar histologic features depending on time of detection	Banff active ABMR C4d positivity and thrombotic microangiopathy usually present. Banff cg = 0	Abrupt allograft dysfunction correlating with increased DSA quantity usually 7–14 days post-transplant
Late (>30 days post-transplant)	Preexisting		Banff active or chronic active ABMR (continuum) +/− C4d positivity	+/− allograft dysfunction and proteinuria Can occur in patients with or without Early active (<30 days post-transplant active ABMR)
	De novo (MOST COMMON)		Banff active or chronic active ABMR (continuum) +/− C4d positivity Concomitant TCMR often present with *de novo* DSA	+/− allograft dysfunction and proteinuria

Hyperacute rejection is a rare in the current era of transplant and occurs minutes to hours post-transplant leading to almost immediate allograft loss. Even if hyperacute rejection is avoided, patients with preexisting DSA at transplant are at risk for an early acute active ABMR <30 days after transplantation during a memory B cell response Table 1. Early active ABMR can occur among patients with a prior sensitizing event (e.g., blood transufions, pregnancy, or prior transplant) without preexisting DSA, but this is less common.

Most cases of active ABMR in current practice are now detected *late* (>30 days post-transplant) and are associated with inferior allograft survival Table 1 (12, 13). Late ABMR >30 days post-transplant now usually develops in the context of *de novo* DSA from underimmunosuppression Table 1 (14). Because these rejections are associated with underimmunosuppression, they are often termed “mixed” and include histologic features of T cell mediated rejection. Unfortunately, concomitant features of T cell mediated rejection are associated with an inferior prognosis (10). Late ABMR in a patient with known preexisting DSA can represent ongoing inflammation and injury after an early acute ABMR episode or can appear later. The onset can be insidious, subclinical, and is often unrecognized without histologic surveillance.

Whether the histology is active or chronic active ABMR depends on the timing of the diagnosis and biopsy. A histologic diagnosis of chronic active ABMR also does not account for the spectrum of activity and chronicity. Cases of chronic active ABMR cases include those with moderate to severe microvascular inflammation with mild chronicity or cases with mild microvascular inflammation with severe chronicity (15). The key message is that the diagnosis of active and chronic active ABMR encompasses a breadth of phenotypes with varied outcomes.

### Understand the baseline risk of ABMR among incompatible kidney transplant recipients

Patients transplanted with known preexisting DSA are at the highest risk of ABMR, but this risk varies based on whether the preexisting DSA was detected with Luminex single antigen bead (SAB) only or if the crossmatch (XM) was positive. Patients with no DSA with SAB testing are at the lowest risk, while patients with SAB and CDC crossmatch positivity are at the highest risk for early active ABMR (<30 days post-transplant), chronic active ABMR, and eventual graft loss. The risk for patients with flow cytometric XM positivity is between those with SAB positivity only and those with CDC XM positivity.

Specifically the risk for early active ABMR is 5%–10% among patients with DSA based on SAB alone and up to 40%–50% when the flow cytometric XM is positive (16, 17). The difference in long term outcomes based on pretransplant and XM positivity has been shown in several studies (18, 19). A large multicenter study by Orandi et al. also clearly showed the stratification of allograft loss based on baseline DSA testing positivity (20).

The effect of incompletely considering these outcome differences was shown in an important industry sponsored multicenter phase 2 randomized controlled trial that examined the efficacy of eculizumab (a terminal C5 complement inhibitor) in reducing the risk of early active ABMR among kidney transplant patients who received a positive XM living donor kidney transplant. A single center nonrandomized study found that the incidence of ABMR was 7.7% among patients treated with eculizumab compared to 41.2% (*p* = 0.0031) among those in the historical control group (17). Unfortunately, a follow-up phase 2 multicenter randomized study observed similar rates of rejection among the groups: 9.8% with eculizumab compared to 13.7% (*p* = 0.760) among controls (21). Close examination of the baseline characteristics may explain why the results from the retrospective study were not replicated. All patients in the single center retrospective study had a positive flow cytometric XM, while lower risk patients were included in the multicenter randomized study. Specifically, 24% of patients in the eculizumab treated group and 35% of controls had negative crossmatch results (21).

## Considerations for outcome assessment

The literature in the ABMR field often refers to patients *responding* to ABMR treatment. What does this mean? Is the response clinically meaningful? Can it be reproduced? The most clinically meaningful outcome measure is allograft survival, but some patients live with ABMR for years. Appropriately powering and conducting clinical trials that use allograft loss as the primary endpoint are expensive and often not feasible. Therefore, defining appropriate clinical endpoints is central to the design of meaningful studies. The next section aims to discuss potential surrogate endpoints and drawbacks to consider.

### Surrogate endpoints

The United States Food and Drug Agency requires that the outcome studied be a well-defined and reliable assessment of how a patient feels, functions, or survives to get a drug approved (22). This ensures that within the stated context of use (e.g., ABMR), the results of an assessment can be relied upon. The FDA allows for using a surrogate endpoint to reduce the duration of follow-up and required sample sizes. Showing efficacy with a validated surrogate endpoint would lead to accelerated approval, but full drug approval would only be granted after showing that the intervention leads to an improvement in the actual endpoint (e.g., graft survival).

Not all endpoints that *correlate* with improved graft survival are appropriate surrogate endpoints. The intervention itself could have mechanisms of action that are independent of its intended effects on the disease process (23). Requirements for a reliable surrogate endpoint can be exceedingly difficult to achieve. Surrogate endpoints must must correlate with the risk of the outcome in patients without any intervention and continue to correlate with the risk of the outcome after intervention. The levels of the surrogate must predict the net effect of the intervention and the correlation between the surrogate endpoint and outcome must be constant across classes of interventions and populations (24). We will discuss the advantages and drawbacks of potential endpoints that have been studied in ABMR trials.

#### Kidney function measures

Graft function as determined by eGFR is commonly selected as a study outcome because of its connection to graft failure. The problem is that the eGFR equations themselves have limitations particularly in kidney transplant patients (25). Estimated GFR can also fluctuate and be affected by multiple factors (e.g., hydration, calcineurin inhibitors, acute kidney injury etc.). In fact, a recent study showed that the trend in serum creatinine does not predict histologic features on a follow-up biopsy (26). Considering these issues, a one-time eGFR measurement or change in GFR at two separate time points is not an acceptable outcome measure as too many factors can influence the result.

In contrast, determining the slope of eGFR decline is a more reliable indicator of the trajectory graft function compared to time point measurements. Estimated GFR trajectories have been incorporated in several models (27). In fact, a multicenter study of 91 patients found that a significant change in eGFR was seen within the first 12 months after active ABMR diagnosis that worsened by a factor of 0.757 ml/min/1.73 m^2 per^ month during a 12-month analysis. It was extrapolated that a 30% improvement in eGFR slope in the first 12 months was associated with a 10% improvement in death-censored graft failure at 5 years (28). This data supported the FDA approval of eGFR slope as a reasonable surrogate endpoint in kidney transplantation trials.

#### Renal histology

The Banff Classification of Allograft Pathology has undoubtedly been a major advance in transplantation (29). Use of the standardized Banff classification is the basis for patient inclusion and has been used for outcome assessment. Despite its strengths, this classification has been shown to be vulnerable to misinterpretation which can affect patient management (30). The main limitations include lack of reproducibility, comparability of Banff scores across constantly evolving versions, and oversimplification of a phenotypically complex and heterogeneous category of rejection.

The poor reproducibility of the Banff system among pathologists is a major issue (31, 32). A recent study revealed that even experienced pathologists lack consistency when interpreting Banff scores (33). The agreement between any two pathologists was poor. For instance, the agreement was between 44.8% and 65.7% for glomerulitis, 44.8%–67.2% for peritubular capillaritis, and 53.7–80.6% for transplant glomerulopathy. This variability in reading individual Banff lesional scores extends to diagnostic categories that include multiple lesion scores (33).

The Banff classification for ABMR has rapidly evolved. The downside of this rapid evolution is that it is sometimes hard to compare studies from different eras. The effect of a changing classification was clearly illustrated recently (34). Removing the “suspicious for ABMR” category in 2017 negatively impacted risk stratification (34). In response, the Banff 2022 classification has added new categories to account for microvascular inflammation (Banff glomerulitis and peritubular capillaritis scores ≥2) without C4d or HLA DSA positivity (8).

We are hopeful that future Banff iterations will leverage digital pathology, artificial intelligence and machine learning tools to permit us to navigate from a categorical classification to a more meaningful, consistent, reproducible, auditable, and continuous scoring system (35, 36). Finally, using novel diagnostic tools such as imaging mass cytometry (37) allows an unprecedented characterization of the immune composition with spatially enriched analysis, providing well-defined targets for personalized therapy.

#### HLA donor specific antibody assessment

**Figure 1:**
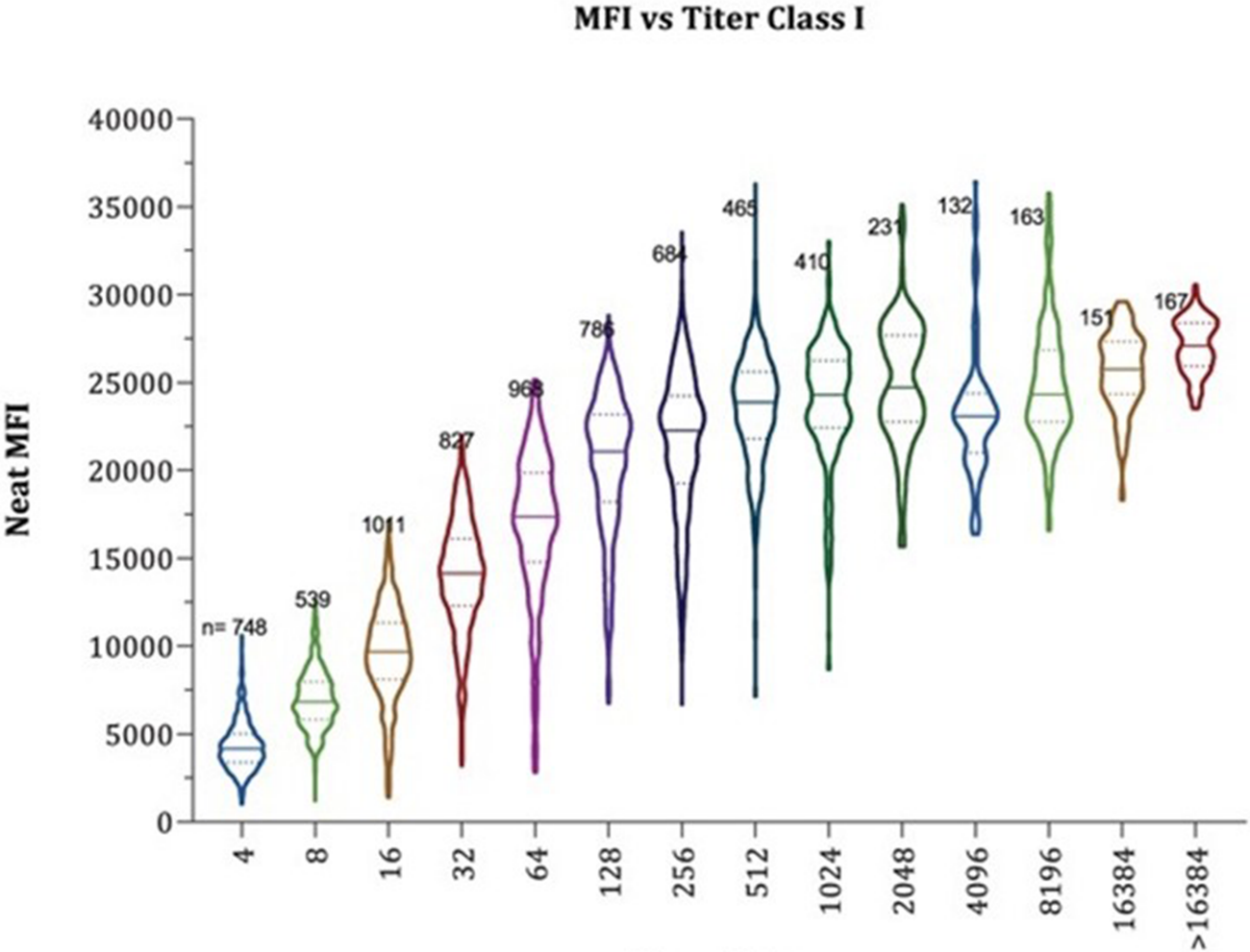
Illustration of the overlap in neat MFI by antibody titer. MFI, mean fluorescence intensity. MFI is a semiquantitative measure of donor specific antibody. As shown in these violin plots, the quantity of donor specific antibody can be vastly different (different titer) despite the same MFI. This emphasizes the point that MFI is not a reliable endpoint for DSA quantification in treatment trials (38). Reprinted with permission from Maguire CH, Schinstock CA, Tambur AR. Measuring human leukocyte antigen alloantibodies: beyond a binary decision. Copyright © 2020 The Author(s) Curr Opin Organ Transplant. (2020) 25(6):p. 529–535 https://journals.lww.com/co-transplantation/fulltext/2020/12000/measuring_human_leukocyte_antigen_alloantibodies_.3.aspx licensed under CC-BY-NC-ND. The Creative Commons license does not apply to this content. Use of the material in any format is prohibited without written permission from the publisher, Wolters Kluwer Health, Inc. Please contact permissions@lww.com for further information.

Measurement of DSA has evolved rapidly. Initial tests were with nonspecific with insensitive CDC crossmatches that were either positive or negative, while modern tests of DSA are based on comprehensive high-resolution donor and recipient HLA typing combined with sensitive and specific SAB solid phase assays that output mean fluorescence intensity (MFI). As described earlier, there appears to be clear correlation with the quantity of DSA and outcomes, but there limitations in our current methods of DSA quantification that affect our ability to use DSA itself as a surrogate endpoint even if one confirms that HLA typing is accurate. Mean fluorescence intensity is a semi quantitative measure that corresponds to the amount of DSA that binds to the antigen on beads -not the true quantity of DSA. There is a maximum number of antigens on these beads. Bead saturation and interference can occur leading to inaccurate results particularly among patients with a high quantity of HLA antibody (38, 39). Performing these solid phase assays with diluted serum to determine antibody titer can provide a better quantitative assessment Figure 1. The neat MFI of multiple DSA specificities was compared to antibody titer and a substantial overlap in MFI was found over a breadth of antibody titer (38). Even if issues of determining DSA quantity are resolved, this will not resolve the challenges of interpreting multiple DSA specificities or varied DSA pathogenicity. Lab to lab variability and insufficient reproducibility further complicate DSA measurement (40). Ensuring accurate HLA typing, investigating the change in DSA with consistent assays, and the use of titers for antibody quantification may be more informative (41, 42).

#### Integrative box (iBox) scoring system

Surrogate endpoints based on one marker (e.g., eGFR) may be insufficient. A composite endpoint comprised of multiple predictive factors may be more predictive of death censored graft failure. The iBox risk prediction tool utilizes a combination of variables including eGFR, proteinuria, HLA DSA, and histology information at 6–24 months to generate individual predictions at 3, 5, and 7 years post transplant (43). This novel tool was developed based on large international multicenter derivation and validation cohorts with more than 10,000 patients. Full and abbreviated (histology omited) iBox systems have been developed with c-statistics ranging from 0.70 to 0.93 in validation cohorts (44). Furthermore, the accuracy of the iBox risk score to predict death censored allograft failure was confirmed in *post hoc* analysis of data from randomized controlled trials in which the iBox scores appeared to change based on treatment (45). The qualification of iBOX as a reasonable likely surrogate endpoint is ongoing with the FDA (46) and the European Medicine Agency (EMA), has approved the iBox for use as a surrogate endpoint to demonstrate the superiority of a new immunosuppressive therapy compared to the standard of care from 6 to 24 months post transplant (44). While a surrogate endpoint comprised of multiple factors may be preferable to the use of a single factor, the limitations of the factors included in the scoring system remain relevant.

#### Molecular tests

Molecular profiling of biopsies at the transcriptomic level is helpful because of its sensitivity, objectivity, and ability to detect early changes (47). As an added benefit, these tests shed light on the pathogenic mechanisms contributing to disease. Most of the published literature historically in the field of rejection concentrated on microarrays obtained on extra biopsy cores stored in RNA later Stabilization solution (Molecular Microscope MMDx One Lambda) (48–50). This revolutionary technology was difficult to apply to practice because of the need for an extra core of sample stored in RNA later. Recently other technologies have been developed using formalin-fixed paraffin-embedded specimens. One example is the Nano String nCounter system (Nano String Technologies, Seattle, WA) which is advantageous because a separate core is not needed, transcripts can be assessed on the same tissue examined visually, and large retrospective studies can be conducted (51–53). This technology is simpler to use and less expensive. While there is promise in these recent technologies, there can be a degree of overlap in the gene transcripts with varied histologic diagnoses (47).

Innovative single cell RNA sequencing and multiplex imaging for transcriptomic and spatial profiling of allograft tissue from patients experiencing different rejection phenotypes are also now available for investigational use. These revolutionary technologies can advance our understanding of the mechanisms of disease because they can help us understand heterogenous immune cell populations in ABMR (37). Spatial profiling has been performed using imaging mass cytometry (37), multiple iterative labeling by antibody neodeposition (50), and the GeoMX digital space profiling platform (51). The studies have highlighted the heterogeneity of the immune landscape and correlated these changes to the kidney microstructures. While innovative molecular technologies are promising and will provide insights on the mechanisms of disease, there is a need for validation before using as endpoints in clinical trials or practice.

#### Donor derived cell free DNA

Donor derived cell free DNA at varied levels can be detected in the peripheral blood of transplant recipients and is increasingly used as a surveillance biomarker of rejection (54). An increased percentage of donor derived cell free DNA correlates with allograft injury (54). The clear advantage of this test is that it is noninvasive. Some researchers have examined the change in the percentage of donor derived cell free DNA with treatment (55). The most obvious challenge with this donor derived cell free DNA is it is only a marker of injury and not specific for ABMR. It also provides little information about what histologic features to expect (e.g., degree of microvascular inflammation or chronicity). This technology is now rapidly expanding. The test is now offered by several companies and local labs will now be running these tests. Thus, there are important unanswered questions about the accuracy, precision, and reproducibility of these tests and laboratory proficiency is needed. This background is necessary before conclusions can be made about the clinical use and validity of these tests to inform.

## Need for collaboration and standardized reporting

Large multicenter, randomized controlled trials are the paradigm for determining the efficacy and safety of new therapies, but applying this approach to transplant and ABMR has proven difficult. The number of patients that meet specific inclusion criteria can be small. One obvious way to overcome this challenge is through the development of research consortia with consistent protocols. Another way of overcoming this challenge is with the adoption of novel trial designs that employ master protocols and Bayesian adaptive approaches that can adapt depending on short-term outcomes (56, 57). These innovative trial designs have already been successful in other area and can be adapted to transplant (58, 59).

Standardized and consistent reporting across centers is essential for research consortia to be effective and for accurate literature interpretation. A recent systematic review of ABMR studies highlighted the lack crucial details on patient inclusion and outcomes. Out of 163 articles reviewed, only 98 reported on XM positivity, while information on sample handling, assays, immunosuppression, treatment, and DSA specificity was inconsistently provided (60). This deficiency hampers understanding, generalization, meta-analysis, and potentially skews results from machine learning algorithms. Clarifying data details is critical to prevent misinterpretation and guide future study design.

## Summary

Improving study design for ABMR presents numerous opportunities despite existing challenges (Table 2). Learning from past experiences is crucial, given the variability in outcomes based on patient, histology, and DSA characteristics. Minimizing or addressing this heterogeneity is vital to prevent unexpected results, although it may reduce sample size and generalizability. Collaboration within the community to establish and validate surrogate endpoints, form consortia, and adopt adaptive clinical trial designs can help overcome these obstacles. Standardizing reporting in retrospective and prospective studies is also essential for accurate communication within the community.

**Table 2:** Challenges and opportunities to improve studies in ABMR.

Challenges	Opportunities
Heterogenous cases with varied clinical outcomes	
• Varied baseline DSA quantity • Preexisting versus *de novo* DSA • ABMR detected via surveillance or indication biopsy	• Plan to enroll patients with a similar risk profile as those included in pilot and early observational studies. • Balance the inclusion of patients with preexisting and *de novo* DSA. Adjust for whether the ABMR diagnosis was made via indication or surveillance biopsy
Difficult to conduct clinical trials because of low enrollment and need for prolonged follow-up	
• The time to graft loss after ABMR detection can be several years. • High risk transplants with DSA and positive crossmatch are done less often making it more difficult to enroll patients in clinical trials. • The downside of improving the homogeneity in the studied patient population is a decrease in patients who meet inclusion criteria. • Patients with chronic ABMR often not found early because these patients may be followed by non-transplant nephrologists and/or do not get surveillance DSA or biopsies	• Develop international consortia. • To account for long follow-up, consider using reliable qualified surrogate endpoints such as slope of eGFR, and plan for long term extension studies to verify results. • Be realistic about enrollment and include centers experienced in transplantation with donor specific antibody. • Develop decentralized clinical trials and partner with local general nephrologists to enroll and identify patients who do not have long term follow-up in an academic medical center. • Consider novel clinical trial design to overcome small patient numbers
Lack of standardized reporting limit the ability to communicate or combine results for meta-analysis	
• Key details about DSA, histology, and patient characteristics often missing in the literature	• Collaboration and development of minimum standards for reporting by major transplant groups (e.g., Banff). • Minimal standard reporting consistently followed by industry and enforced by major clinical journals

## Data Availability

The original contributions presented in the study are included in the article/Supplementary Material, further inquiries can be directed to the corresponding author.
